# Supplementary material for: Passive Immunity Trial for Our Nation (PassITON): study protocol for a randomized placebo-control clinical trial evaluating COVID-19 convalescent plasma in hospitalized adults
Source: Res Sq. 2021 Mar 2:rs.3.rs-227796. Preprint. [Version 1] doi: 10.21203/rs.3.rs-227796/v1 (PMC7941637; doi:10.21203/rs.3.rs-227796/v1)
Supplement: Supplement [file 046a2e5f28db6dbf1b61df12.pdf]

Passive Immunity Trial for Our Nation (PassItOn)

STATISTICAL ANALYSIS PLAN (SAP)

Acronym:	PassItOn
Funder:	Dolly Parton COVID-19 Research Fund and the National Center for Advancing Translational Sciences (NCATS) of the National Institutes of Health (NIH)
SAP version	1.0
Date:	12 December 2020
Statisticians	Thomas G. Stewart, PhD<br>Christopher J. Lindsell, PhD
Principal Investigators	Todd W. Rice, MD, MSc<br>Wesley H. Self, MD, MPH
Program Manager	Jillian Rhoads, PhD
Safety Monitor	Jonathan D. Casey, MD
Coordinating Center	Vanderbilt University Medical Center

Table of Contents

1. Summary ... 4

1.1 General Approach ... 6

2. Planned interim analyses for mortality ... 7

3. Analysis plan for primary endpoint ... 8

3.1 Model fit ... 9

3.2 Missing data ... 9

3.3 Planned Sensitivity Analyses ... 9

4. Type I error rate and power ... 9

5 Analysis plan for additional analyses of the primary endpoint ... 10

5.1 Quantification of donor plasma efficacy ... 10

5.2 Effect modification analyses of the primary outcome ... 11

6 Analysis plan for secondary endpoints ... 11

6.1 Ordinal outcomes ... 12

6.2 Binary outcomes ... 12

6.3 Time to event outcomes ... 12

6.4 Outcomes with a competing risk of death ... 12

7 Safety Outcomes and Adverse Events ... 12

8 Definition of the Primary Outcome ... 13

9 Definition of SOFA Score ... 13

10 Definition of Secondary Outcomes ... 14

10.1 All-location, all-cause 14-day mortality ... 14

10.2 All-location, all-cause 28-day mortality ... 14

10.3 Survival through 28 days ... 14

10.4 Hospital discharge through 28 days ... 15

10.5 Time to Recovery ... 15

10.6 COVID Ordinal Outcomes Scale on Study Day 3 ... 15

10.7 COVID Ordinal Outcomes Scale on Study Day 8 ... 15

10.8 COVID Ordinal Outcomes Scale on Study Day 29 ... 15

10.9 Days Free of Support ... 16

10.9.1 Oxygen-free days through Day 28 16
10.9.2 Ventilator-free days to Day 28.......... 16
10.9.3 Vasopressor-free days to Day 28 17
10.9.4 ICU-free days to Day 28 17
10.9.5 Hospital-free days to Day 28.......... 17
11 Definition of Safety Outcomes.......... 18
11.1 Acute kidney injury 18
11.2 Receipt of renal replacement therapy 18
11.3 Documented venous thromboembolic disease (DVT or PE) 18
11.4 Documented cardiovascular event (myocardial infarction or ischemic stroke) 18
11.5 Transfusion reaction.......... 18
11.6 Transfusion related acute lung injury (TRALI).......... 19
11.7 Transfusion associated circulatory overload (TACO) 19
11.8 Transfusion related infection.......... 19
12 Literature Cited

1. Summary

The key features of the trial are summarized in detail in the study protocol. The following table restates some of the key study features from the protocol.

Feature	Description
Study Design	Multicenter, blinded, placebo-controlled randomized clinical trial
Randomization	Eligible participants will be randomized 1:1 to convalescent plasma versus lactated Ringer's solution with multivitamins. Randomization will be completed in permuted blocks and stratified by site, gender, and age.
Blinding	Patients and outcome assessors will be blinded to group assignment.
Intervention group treatment	1 unit (200–399 ml) of SARS-CoV-2 convalescent plasma infused intravenously
Control group treatment	250 ml of Lactated Ringer's with multivitamins, which visually resembles plasma, infused intravenously
Inclusion Criteria	1. Age greater than or equal to 18 years of age<br>2. Currently hospitalized or in an emergency department with anticipated hospitalization<br>3. Symptoms of acute respiratory infection, defined as one or more of the following:<br>a. Cough<br>b. Chills, or a fever (greater than 37.5° C or 99.5° F)<br>c. Shortness of breath, operationalized as a patient having any of the following:<br>i. Subjective shortness of breath reported by a patient or surrogate.<br>ii. Tachypnea with respiratory rate of greater than 22 breaths per minute<br>iii. Hypoxemia, defined as SpO2 less than 92% on room air, new receipt of supplemental oxygen to maintain SpO2 greater than or equal to 92%, or increased supplemental oxygen to maintain SpO2 greater than or equal to 92% for a patient on chronic oxygen therapy<br>4. Laboratory-confirmed SARS-CoV-2 infection within the past 14 days
Exclusion Criteria	1. Prisoner<br>2. Unable to randomize within 14 days after onset of acute respiratory infection symptoms<br>3. Patient, legal representative, or physician not committed to full support (Exception: a patient who will receive all supportive care except for attempts at resuscitation from cardiac arrest will not be excluded.)<br>4. Inability to be contacted on Day 29-36 for clinical outcome assessment<br>5. Receipt of COVID-19 convalescent plasma or pooled immunoglobulin in the past 30 days<br>6. Contraindications to transfusion or history of prior reactions to transfused blood products

	7. Plan for hospital discharge within 24 hours of enrollment<br>8. Previous enrollment in this trial<br>9. Enrollment in another clinical trial evaluating monoclonal antibodies, convalescent plasma, or another passive immunity therapy
Sample Size	1000
Primary Outcome	COVID-19 7-point Ordinal Clinical Progression Outcomes Scale on Study Day 15:<br>1. Not hospitalized with resumption of normal activities.<br>2. Not hospitalized, but unable to resume normal activities.<br>3. Hospitalized, not on supplemental oxygen.<br>4. Hospitalized, and on supplemental oxygen.<br>5. Hospitalized, on nasal high-flow oxygen therapy, noninvasive mechanical ventilation, or both<br>6. Hospitalized, on ECMO, invasive mechanical ventilation, or both.<br>7. Death
Secondary Outcomes	• All-location, all-cause 14-day mortality (assessed on Study Day 15)<br>• All-location, all-cause 28-day mortality (assessed on Study Day 29)<br>• Survival through 28 days<br>• Time to hospital discharge through 28 days<br>• Time to recovery (defined as time from randomization to the earlier of final oxygen receipt in the hospital or hospital discharge).<br>• COVID-19 7-point Ordinal Clinical Progression Outcomes Scale on Study Day 3, 8, and 29<br>• Oxygen-free days through Day 28<br>• Ventilator-free days through Day 28<br>• Vasopressor-free days through Day 28<br>• ICU-free days through Day 28<br>• Hospital-free days through Day 28<br>• Endpoints developed in the course of the pandemic
Safety Outcomes	• Acute kidney injury<br>• Receipt of renal replacement therapy<br>• Documented venous thromboembolic disease (DVT or PE)<br>• Documented cardiovascular event (myocardial infarction or ischemic stroke)<br>• Transfusion reaction<br>• Transfusion related acute lung injury (TRALI)<br>• Transfusion associated circulatory overload (TACO)<br>• Transfusion related infection
Primary AIM	Assess if the treatment intervention improves the day 15 endpoint compared to the control arm<br><br>It is hypothesized that among adults hospitalized with COVID-19, administration of convalescent plasma will improve clinical status 14 days after randomization.

Inference approach	The primary point of inference will be the likelihood ratio and corresponding support interval for the covariate adjusted treatment effect odds ratio. Likelihood ratios more extreme than 7 (or 1/7) will be interpreted as sufficient evidence to assert efficacy.<br><br>The odds ratio will be estimated with a cumulative probability ordinal regression model with logit link.
Planned Interim Analyses	There are 3 interim analyses for safety endpoints, and a final analysis of efficacy endpoints. The safety interim analyses will occur after 15 days of follow-up is completed for the first 150, 450, and 750 patients. The final analysis will occur after follow-up and data lock is complete for all enrolled subjects.
Planned Reporting Triggers	There are no planned reporting triggers.
Planned stopping rules	The trial will end for safety if a one-sided test of the difference in risk of mortality between control and intervention arms is significant at the alpha = 0.1 level. The mortality stopping rule will be evaluated at the 3 interim analyses described above.
Type I error rate	The combination of a 1/7 likelihood ratio threshold and the safety stopping rule result in a simulated type I error rate of 0.02
Power	The minimal detectable effect at 80% power is an OR of 0.73.

1.1 General Approach

The final statistical design for this trial was informed by the need to learn as rapidly as possible from the data during the pandemic while simultaneously managing the risk of drawing erroneous conclusions. Additionally, the DSMB requested that the analysis plan be modified to reduce the number of interim analyses and to anticipate the possibility that the study might need to enroll a larger number of subjects than initially proposed. This requires a framework for decision-making that does not demand that all possible looks of the data are prespecified, as is required of approaches based on p-values. Two approaches which offer the needed flexibility are the likelihood and Bayesian frameworks, which are closely related. The likelihood approach uses the likelihood function to generate point-estimates and interval estimates of the treatment effect. Moreover, competing hypotheses are compared with likelihood ratio (LR), to measure the relative strength of evidence for the null and alternative hypotheses. Because it retains its meaning and reliability regardless of the number of looks at the data or endpoints under consideration[1,2], the LR has been successfully implemented in clinical trials, including in trials with continuous monitoring or sequential methods.[3,4] The closely related Bayesian framework has also been successfully implemented in COVID-19 clinical trials.[5] The Bayesian framework combines the likelihood function with a researcher prior distribution in order measure the strength of evidence for competing hypotheses on an absolute, probability scale rather than the relative scale of the likelihood approach. We acknowledge that the Bayesian approach could have also been implemented as a decision-making framework in this study (and similar studies for which the schedule of interim analyses is not fixed). However, to avoid the ambiguous task of eliciting a prior distribution, we opted to focus on the likelihood alone.

Decision making using the likelihood approach in a clinical trial centers on three quantities: the point estimate of the treatment effect (an odds ratio, for example), a corresponding interval estimate, and a single number summary---the likelihood ratio---which measures the relative evidence for one hypothesis compared to another. These three quantities are not unlike the point estimate, 95% confidence interval, and p-value that are generated in frequentist analyses. In fact, the point estimates of often identical, and the interval estimates are often similar. The LR and the p-value, however, are distinct measures of evidence. The LR is a ratio: the density of the trial data if the treatment works (alternative hypothesis) divided by the density of the trial data if the treatment does not work (null hypothesis). A LR of 1 indicates the data are neutral; neither hypothesis is supported over the other. Large LR are evidence in support of the treatment working while small LR are evidence in support of the null. In short, the LR level of evidence is based on comparing the likelihood of the data under two competing models – the alternative hypothesis and the null hypothesis. This is different to using a p-value as the level of evidence because the p-value essentially compares what actually happened in the trial to what might have happened in the trial if it were repeated infinitely and the null hypothesis were true. Because it is impossible to compute what might have happened if the rules for decision making are not fully predefined, using a p-value for decision making is not well suited for a trial like this one in which DSMB requests and pandemic circumstances prompted design changes. The LR approach on the other hand, is based on a relative likelihood of observed outcomes under two competing models at the same point in time making it especially appropriate for settings where pre-specification of the timing or frequency of sequential analyses is not possible. In this study, a LR of 7 or larger in favor of treatment is considered sufficient evidence to assert that the treatment is beneficial.

Even though the LR (instead of the p-value) is the quantity of primary interest for decision making, the analysis plan does control type 1 error. Sequential likelihood ratio tests of two prespecified hypotheses have a natural bound on type 1 error of 1/k where k is the threshold for asserting efficacy. In this study, the combination of the stopping rule for mortality and the large number of subjects accrued before the first analysis for efficacy result in a bound on type 1 error well within traditional levels of 0.05 as described in detail below.

2. Planned interim analyses for mortality

Initially, this trial included frequent interim analyses with reporting triggers for efficacy, harm, and futility. At DSMB request, the frequency of interim analyses was reduced, and reporting triggers were replaced with a single stopping rule for harm based on a comparison of day 15 mortality between placebo and intervention arms. While the DSMB requested that a flexible sample size be accommodated in our design, they did not request formal rules for extending the study because the context for decision making in a pandemic is unknown, further emphasizing the utility of the LR approach.

The three interim analyses are planned to occur after day 15 follow-up data collection is completed for 150, 450, and 750 study subjects. Adverse events, safety outcomes, protocol deviations, and the primary endpoint will be described grouped by study arm. Initially, the intervention arm and placebo labels will be replaced with generic labels “treatment A” and “treatment B”, and the DSMB Chair may request to unmask the group assignment.

The difference in mortality risk will be calculated, and the one-sided hypothesis that mortality risk in the intervention arm exceeds the mortality risk in placebo will be compared to the null hypothesis of equal mortality risk.

	Hypothesis	
Null	$\Delta \leq 0$	(Treatment is beneficial or neutral)
Alternative	$\Delta > 0$	(Treatment is harmful).
$\Delta = P(\text{mortality among treated}) - P(\text{mortality among controls})$		

The trial will be stopped if the likelihood ratio exceeds the threshold listed below. Generally, the level of evidence required to stop a study for safety concerns is less than the level of evidence required to assert efficacy of the intervention. Likewise, there is not the same level of concern for type 1 error control for safety outcomes as there is for efficacy endpoints; however, the threshold values were selected so that the trial-wise risk of ending early for mortality is 0.1 if the treatment is equivalent to placebo ($\Delta = 0$). To provide context for the LR thresholds listed in the table below, we have also provided α_N, the p-value threshold which approximates the LR threshold if the p-value were calculated from a likelihood ratio test.

N	LR Threshold	α_N
150	6.3	0.0275
450	4.0	0.0479
750	3.3	0.0612

3. Analysis plan for primary endpoint

The data from the clinical trial will be analyzed after follow-up and data lock is completed for all study subjects. The analysis will be intent-to-treat, meaning that patients will be analyzed according to the randomization schedule regardless of the treatment administered. The main result will be an estimate of the treatment effect odds ratio, its likelihood ratio when compared to the null, and the corresponding 1/7 likelihood support interval, all of which will be estimated from a cumulative probability ordinal regression model (CPM) with logit link. The marginal likelihood function for the treatment effect parameter will be the asymptotic regression coefficient distribution; specifically, it will be the normal distribution density function with mean and standard deviation equal to the regression estimates. Likelihood ratios more extreme than 7 will be interpreted as sufficient evidence to assert efficacy. In order to increase study power, the following variables will also be included into the regression:

1. Treatment (1 parameter)
2. Age (2 parameters, restricted cubic spline)
3. Sex (1 parameter)
4. Baseline SOFA score (1 parameter, linear term)
5. Baseline COVID-19 7-point Ordinal Clinical Progression Outcomes Scale score (possible range:3-6) (2 parameters, quadratic)
6. Time from symptom onset in days (2 parameter, non-linear term)
7. Site indicator variables (either as a main effect or as a random effect, depending on the number of sites enrolling patients)

3.1 Model fit

Model fit will be assessed with probability scale residuals and with leave-one-out diagnostics like DFBETAS. If model diagnostics indicate that an alternative link function provides a superior fit, then that alternative link function will be proposed instead.

3.2 Missing data

We anticipate rare or no missingness for data points during the patients' in-hospital course. There may be missing data for the COVID-19 7-point Ordinal Clinical Progression Outcomes Scale score after hospital discharge, which relies on phone follow-up. After discharge, distinguishing between category 1 and category 2 on the scale requires speaking to the participant or surrogate about normal daily activities. If the patient is not reached for follow-up phone calls after discharge, the COVID-19 7-point Ordinal Clinical Progression Outcomes Scale score will be classified as category 2 (not hospitalized, but unable to resume normal activities). If the patient is reached for a follow-up call post-charge and reports category 1, this classification of category 1 will be carried forward to future follow-up calls if the patient cannot be reached. For example, if the patient cannot be reached for the Day 8, 15, or 29 follow-up calls, a category 2 score will be reported for all three visits. If the patient is successfully contacted on the Day 8 call and reports category 1, and then the patient cannot be reached for a Day 15 call, the category 1 score from Day 8 will be carried forward to Day 15.

Missing data for a covariate used in the primary model will be multiply imputed using predictive mean matching if number of observations missing covariate values exceeds 5 percent. If fewer than 5% of observations have missing covariate values, missing values will be imputed with a single conditional mean.

3.3 Planned Sensitivity Analyses

In addition to the primary as-assigned analysis of the endpoint, a per-protocol analysis of the primary endpoint will be performed in which patients that failed to start their assigned study transfusion in compliance with the protocol will be excluded. An as-exposed analysis will not be performed.

4. Type I error rate and power

The long-run operating characteristics were estimated by generating study data to reflect different treatment effect sizes. The simulated study dataset was evaluated according to the stopping rule and analysis plan described above. For each effect size, 1000 simulated datasets were analyzed. A Type I error occurred if the study asserted efficacy when in fact there was no treatment effect. For each treatment effect, the Type I error rate was calculated as the proportion of null-effect studies in which the error occurred. A Type II error occurred if the study failed to assert efficacy when there was a beneficial treatment effect. For each treatment effect, power was calculated as the proportion of studies that did not result in a Type II error.

The study endpoint for control subjects was simulated to match the outcomes in the control arm of a recent clinical trial.[6] In each simulation setting, the distribution for the treatment arm was calculated by adjusting the control arm outcome distribution according to the setting-specific treatment effect size and data generation model.

These simulations demonstrated that enrollment of 1000 patients (500 patients in the intervention group and 500 patients in the control group) would provide 80% power to detect an adjusted odds ratio of ≤0.73. Some trials orient the ordinal outcomes scale in the reverse direction, with an odds ratio greater than 1.0 indicating benefit from the intervention. With reversal of the ordinal outcomes scale, enrollment of 1000 patients would provide 80% power to detect an adjusted odds ratio ≥1.37. The simulations also demonstrated that the type I error rate was bounded below 0.05. The figure below is the estimated power curve for the trial design.

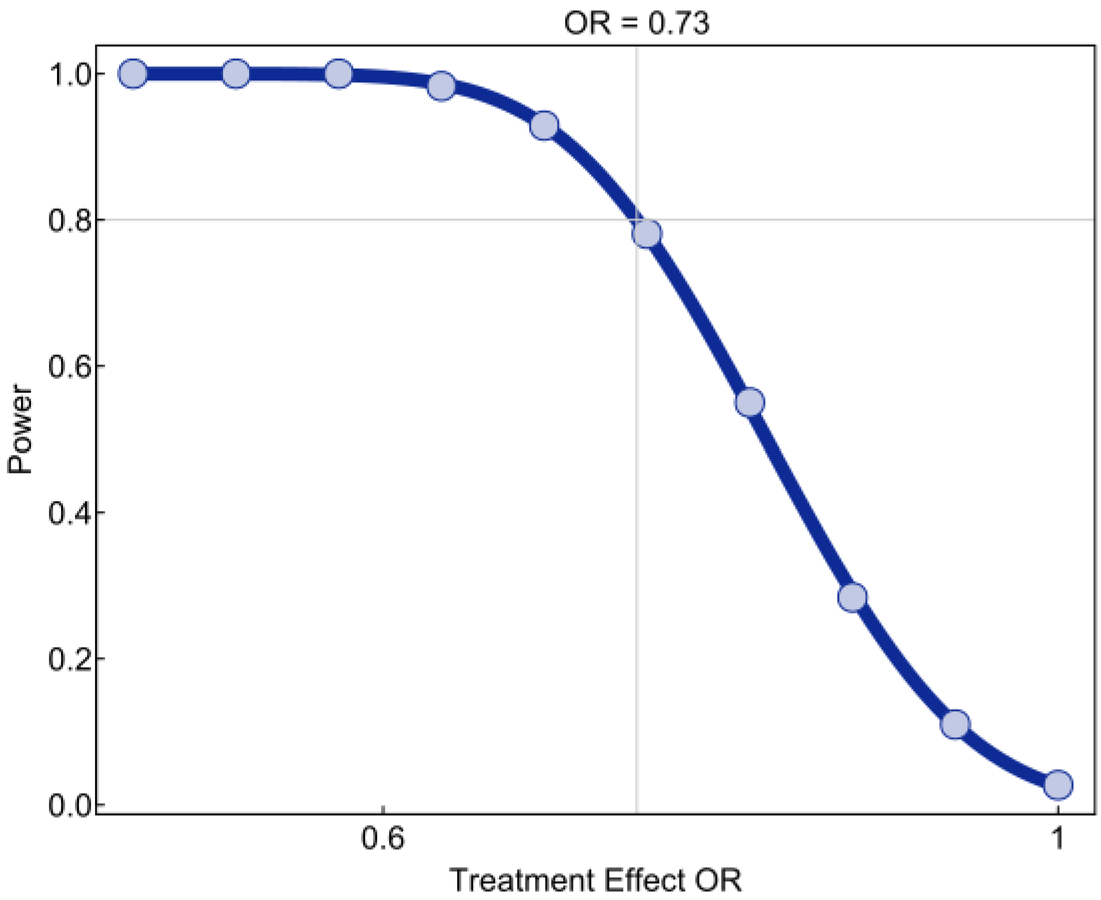

5 Analysis plan for additional analyses of the primary endpoint

5.1 Quantification of donor plasma efficacy

Quantification of donor plasma efficacy. The impact of the binding and neutralization levels of the donor plasma on the primary endpoint will be estimated with two ordinal regression models. In the first, the model will include the same covariates listed for the primary endpoint analysis with the addition of a measure of donor plasma binding level. Similarly, in the second model, a measure of donor plasma

neutralization levels will be added to the models. The variables will be included into the models as a restricted cubic spline with three knots in order to capture potential non-linear associations with the outcome. For observations in the control arm, binding and neutralization values will be set to zero.

5.2 Effect modification analyses of the primary outcome

The degree to which pre-specified baseline variables modify the treatment effect will be examined with tests of statistical interaction in a proportional odds regression model. Independent variables will include study group assignment, the potential effect modifier of interest, the interaction between the two, and the same pre-specified covariates used in the primary model. Presence of effect modification will be assessed by reference to the LR for the interaction term, with values greater than 6 considered to suggest a potential interaction and values greater than 7 considered to confirm an interaction.

We will examine whether the following pre-specified baseline variables modify the effect of study group on the primary outcome:

- Baseline recipient (trial participant) antibody quantification
- Baseline COVID scale
- Baseline SOFA
- ICU/ward enrollment location
- Age
- Race/ethnicity
- Duration of symptoms prior to randomization
- Mechanical ventilation status at baseline

6 Analysis plan for secondary endpoints

The analyses for secondary endpoints will be intent-to-treat. The following is a table of secondary endpoints and a column indicating the planned analysis.

Outcome	Type	Analysis
All-location, all-cause 14-day mortality (assessed on Study Day 15)	Binary	Logistic regression
All-location, all-cause 28-day mortality (assessed on Study Day 29)	Binary	Logistic regression
Survival through 28 days	Time-to-event	Proportional hazards regression
Time to hospital discharge through 28 days	Time-to-event	Multistate model with death as a competing risk

Time to recovery (defined as time from randomization to the earlier of final oxygen receipt in the hospital or hospital discharge)	Time-to-event	CPM with logit link
COVID-19 7-point Ordinal Clinical Progression Outcomes Scale on Study Day 3, 8 and 29	Ordinal	CPM with logit link
Oxygen-free days through Day 28	Ordinal	CPM with logit link
Ventilator-free days through Day 28	Ordinal	CPM with logit link
Vasopressor-free days through Day 28	Ordinal	CPM with logit link
ICU-free days through Day 28	Ordinal	CPM with logit link
Hospital-free days through Day 28	Ordinal	CPM with logit link

6.1 Ordinal outcomes

Ordinal secondary outcomes will be analyzed using the same model described for the primary endpoint. Similar steps to evaluate model fit and overly influential observations will be performed.

6.2 Binary outcomes

Binary secondary outcomes will be analyzed using multivariable logistic regression models with the same pre-specified covariates as the primary endpoint. To assess model calibration and overly influential observations, graphical displays of calibration and DFBETAS will be created.

6.3 Time to event outcomes

Time to event outcomes will be analyzed with a proportional hazards regression model. The key result of the analysis is an estimate of the treatment effect hazards ratio. The same set of covariates used for the adjusted analysis of the primary endpoint will also be included in the analysis of secondary time-to-event endpoints. Model fit will be assessed with Schoenfeld residuals and with leave-one-out diagnostics like DFBETAS. The proportional odds assumption will be evaluated with graphical displays. Deviations from proportionality will trigger sensitivity analyses.

6.4 Outcomes with a competing risk of death

Some secondary outcomes, like the primary outcome, incorporate death as part of the scale. Others, such as length of hospital stay, do not. Because death censors length of stay, it is a competing outcome. Outcomes with a competing risk of death will be analyzed with a multi-state model. Both the instantaneous risk and cumulative risk of the outcome will be reported.

7 Safety Outcomes and Adverse Events

The frequency and description of safety outcomes and adverse events will be reported for all enrolled patients grouped by treatment assignment. The association between treatment received and safety

outcomes between groups will be estimated without covariate adjustment. The following table lists the safety outcomes and the planned analyses.

Outcome	Type	Analysis
Receipt of renal replacement therapy	Binary	Risk difference
Documented venous thromboembolic disease (DVT or PE)	Binary	Risk difference
Documented cardiovascular event (myocardial infarction or ischemic stroke)	Binary	Risk difference
Transfusion reaction	Binary	Risk difference
Transfusion related acute lung injury (TRALI)	Binary	Risk difference
Transfusion associated circulatory overload (TACO)	Binary	Risk difference
Transfusion related infection	Binary	Risk difference

8 Definition of the Primary Outcome

The primary outcome is patients' clinical status 14 days after randomization (measured on Study Day 15) as assessed with the seven-category COVID Ordinal Outcome Scale:

- Not hospitalized without limitation in activity
- Not hospitalized with limitation in activity
- Hospitalized not on supplemental oxygen
- Hospitalized on supplemental oxygen
- Hospitalized on non-invasive ventilation or high-flow nasal cannula
- Hospitalized on invasive mechanical ventilation or extracorporeal membrane oxygenation
- Death

While patients are in the hospital, primary outcome assessment will be performed by review of the electronic health records. For patients discharged prior to 14 days after randomization, study staff will call patients or their surrogates to obtain the patients' clinical status for primary outcome assignment.

9 Definition of SOFA Score

The SOFA score at baseline (pre-randomization) will used as a co-variable in the regression models for the primary and secondary outcomes. The SOFA score will be calculated using the definitions based on the Third International Consensus Definitions for Sepsis and Septic Shock (Sepsis-3). The SOFA score will be calculated using data available in the 24 hours prior to randomization. We record the following data for SOFA score calculation: lowest SpO2 and FIO2 at the time of the lowest SpO2 measurement; lowest platelet count; highest total bilirubin concentration; lowest Glasgow Coma Score; lowest mean arterial pressure; receipt and dose of inotropes/vasopressors, including dobutamine, dopamine, epinephrine, norepinephrine, phenylephrine, and vasopressin; highest creatinine; highest INR.

For calculation of the respiratory component of the SOFA score, if recorded, FiO2 will be used in the calculation of the S:F ratio. If no FIO2 is recorded, FiO2 will be estimated with the following equation: 0.21 + (supplemental oxygen flow rate in liters/minute) * 0.03. Respiratory SOFA score will be classified by SpO2 and SpO2:FiO2 ratio according to the table below based on the validated technique described by Pandharipande:

**Respiratory SOFA Score assignment based on SpO_2 and FIO_2 values when no PaO2 is recorded**		
**Values**		**Respiratory SOFA Score Assignment**
SpO_2 ≥95% and FiO_2=0.21 (room air)		0
If SpO_2 <95% or FiO_2 >0.21, calculate the SpO_2:FiO_2 ratio and assign respiratory SOFA score based on the thresholds below:		
	SpO_2: FiO_2 ≥357	1
	SpO_2: FiO_2 ≥214 and <357	2
	SpO_2: FiO_2 ≥89 and <214	3
	SpO_2: FiO_2 <89	4

When no S:F ratio is available, the respiratory SOFA score will be imputed as the median observed value in the patients in the trial with non-missing values.

10 Definition of Secondary Outcomes

10.1 All-location, all-cause 14-day mortality

All location, all cause 14-day mortality is defined as death between randomization and 14 days after randomization from any cause and in any location, including after discharge from the index hospitalization. Data on death will be collected from the electronic health record during the hospitalization and then from telephone calls to the patient or surrogate after discharge on Study Day 8, 15 and 29.

10.2 All-location, all-cause 28-day mortality

All location, all cause 28-day mortality is defined as death between randomization and 28 days after randomization from any cause and in any location, including after discharge from the index hospitalization. Data on death will be collected from the electronic health record during the hospitalization and then from telephone calls to the patient or surrogate after discharge on Study Day 8, 15 and 29.

10.3 Survival through 28 days

Vital status will be recorded between randomization and 28 days following randomization using in-hospital data and follow-up calls post-discharge on Study Day 8, 15, and 29.

10.4 Hospital discharge through 28 days

The count of days after randomization until discharge from the index hospitalization occurs, or day 28, whichever is longer, will be recorded. Death will be treated as a competing risk.

10.5 Time to Recovery

Recovery is defined as reaching category 1, 2, or 3 on the seven-category COVID Ordinal Outcome Scale. A patient can reach category 1, 2, or 3 on the seven-category COVID Ordinal Outcome Scale either by (a) being discharged from the hospital or by (b) being liberated from supplemental oxygen for the final time during the index hospitalization. Time to recovery is defined as the time between randomization and the first of either hospital discharge or liberation from supplemental oxygen for the final time during the index hospitalization. Time to recovery is an “in-hospital outcome” and does not reflect information on receipt of supplemental oxygen, rehospitalization, or death after discharge from the index hospitalization.[7]

For a patient who is not receiving supplemental oxygen at the time of randomization, and who never receives supplemental oxygen during the index hospitalization, the time to recovery is 0.0 days. For a patient who receives supplemental oxygen at any time between randomization and discharge from the index hospitalization, but who is not receiving supplemental oxygen at the time of discharge from the index hospitalization, the time to recovery is the time from randomization to the time of final receipt of supplemental oxygen during the index hospitalization. For a patient who is receiving supplemental oxygen at the time of discharge from the index hospitalization, the time to recovery is the time from randomization to the time of discharge from the index hospitalization. Patients who remain hospitalized and receiving supplemental oxygen 28 days after randomization are not considered to have experienced recovery. All patients who die prior to discharge from the index hospitalization are not considered to have experienced recovery. Patients who do not experience recovery will be awarded a value for the outcome of time to recovery of 28.0 days.

10.6 COVID Ordinal Outcomes Scale on Study Day 3

This outcome is defined as clinical status 2 days after randomization (measured on Study Day 3) as assessed with the seven-category COVID Ordinal Outcome Scale, as described in the section on the Primary Outcome. Data on clinical status is collected from the electronic health record during the hospitalization. Patients discharge prior to Study Day 3 will have the Day 3 COVID Ordinal Outcome Scale coded as level 2 (not hospitalized with limitation in activity).

10.7 COVID Ordinal Outcomes Scale on Study Day 8

This outcome is defined as clinical status 7 days after randomization (measured on Study Day 8) as assessed with the seven-category COVID Ordinal Outcome Scale, as described in the section on the Primary Outcome. Data on clinical status is collected from the electronic health record during the hospitalization and then from telephone calls to the patient or surrogate after discharge from the index hospitalization.

10.8 COVID Ordinal Outcomes Scale on Study Day 29

This outcome is defined as clinical status 28 days after randomization (measured on Study Day 29) as assessed with the seven-category COVID Ordinal Outcome Scale, as described in the section on the Primary Outcome. Data on clinical status were collected from the electronic health record during the hospitalization and then from telephone calls to the patient or surrogate after discharge from the index

hospitalization. Missing data for the COVID Ordinal Outcomes Scale on Day 29 will be handled in the same fashion as describe for the primary outcome.

10.9 Days Free of Support

For all outcomes related to days alive and free from a supportive therapy, "-free days" will be calculated as 28 minus the number of days the number of days on support. If a patient is on support for a portion of a day, the whole day is counted as a day on support. The day of randomization contribute to the count of "-free days". Days between randomization and the first receipt of the supportive therapy and days following the last day of the support therapy both count toward the total number of "-free days". Days alive and free of the supportive therapy that occur between periods receiving support do not count toward "-free days". Information on organ support therapies (oxygen, ventilation, vasopressors, ICU care) are collected during the index hospitalization ("in-hospital data") while death is collected both in-hospital and out-of-hospital to Study Day 29 ("all-location data"). Thus, patients who died at any time before Study Day 29 will be coded as having zero-free days.

10.9.1 Oxygen-free days through Day 28

For calculation of oxygen-free days, supplemental oxygen is defined as oxygen administered by nasal cannula, face mask, high-flow nasal cannula, non-invasive ventilation, or invasive ventilation. Positive airway pressure (CPAP, BiPAP) provided solely at night as treatment for sleep-disordered breathing (e.g. obstructive sleep apnea) is not considered supplemental oxygen. Oxygen-free days are calculated as the number of whole calendar days alive and not receiving supplemental oxygen between randomization and study day 29. Patients who die before Day 29 receive a value of 0. If a patient survives through study day 29 and never receives supplemental oxygen, the number of oxygen-free days is 28. If a patient receives supplemental oxygen and survives through study day 28, the number of oxygen free-days is calculated as 28 minus the number of calendar days from the first day on which the patient receives supplemental oxygen until the last day on which the patient receives supplemental oxygen. Days on which the patient does not receive supplemental oxygen that occur between days on which the patient receives supplemental oxygen do not count towards the number of oxygen free days. Data on oxygen use are censored at hospital discharge and the last observed status will be carried forward ("in-hospital outcome"). That is, if the patient is receiving supplement oxygen at hospital discharge, the analysis assumes they continue to receive supplemental oxygen through Study Day 29. If the patient is not receiving supplemental oxygen at hospital discharge, the analysis assumes the patient continues not to receive supplemental oxygen through Study Day 29.

10.9.2 Ventilator-free days to Day 28

Ventilator-free days to day 28 is defined as the number of whole calendar days alive and breathing without invasive mechanical ventilation from 00:00 on the day of randomization through Study Day 29. Patients who die before Study Day 29 receive a value of 0. If a patient survives to the first of discharge or Study Day 29 and never receives invasive mechanical ventilation, the number of VFDs is 28. If a patient receives invasive mechanical ventilation and survives to the first of hospital discharge or Study Day 28, the number of VFDs is calculated as 28 minus the number of calendar days from the first day on which the patient received invasive mechanical ventilation until the last day on which the patient received invasive mechanical ventilation. Days on which the patient does not receive invasive mechanical ventilation that occur between days on which the patient did receive mechanical ventilation do not count towards the number of VFDs. Data on ventilation are censored at hospital discharge and the last observed status is carried forward ("in-hospital outcome"). That is, if the patient is receiving invasive mechanical ventilation at hospital discharge, the analysis assumes s he/she continued to receive invasive mechanical

ventilation through Study Day 29. If the patient is not receiving invasive mechanical ventilation at hospital discharge, the analysis assumes the patient continued not to receive invasive mechanical ventilation through Study Day 29.

10.9.3 Vasopressor-free days to Day 28

For vasopressor-free days, a day on which a patient received of any of the following medications via intravenous drip or push at any dose is considered a day receiving vasopressors: norepinephrine, epinephrine, vasopressin, phenylephrine, angiotensin II, dobutamine, or dopamine. Vasopressor-free days to Day 28 is calculated as the number of whole calendar days alive and not receiving intravenous vasopressors or inotropes from 00:00 on the day of randomization through study day 29. Patients who die before Study Day 29 receive a value of 0. If a patient survives to Study Day 29 and never receives intravenous vasopressors or inotropes, the number of vasopressor-free days is 28. If a patient receives intravenous vasopressors or inotropes and survives to the first of discharge or Study Day 29, the number of vasopressor-free days was calculated as 28 minus the number of calendar days from the first day on which the patient received vasopressors or inotropes until the last day on which the patient received vasopressors or inotropes. Days on which the patient did not receive vasopressors or inotropes that occurred between days on which the patient received vasopressors or inotropes do not count towards the number of vasopressor-free days. Data on vasopressor use is censored at hospital discharge and the last observed status is carried forward ("in-hospital outcome"). That is, if the patient is known to be receiving vasopressors or inotropes at hospital discharge, the analysis assumes he/she continued to receive vasopressors or inotropes through Study Day 29. If the patient was not receiving vasopressors or inotropes at hospital discharge, the analysis assumes the patient continued not to receive vasopressors or inotropes through Study Day 29.

10.9.4 ICU-free days to Day 28

Intensive care unit-free days (ICU-free days) to day 28 is defined as the number of whole calendar days alive and not admitted to an intensive care unit from 00:00 on the day of randomization through Study Day 29. Patients who die before Study Day 29 receive a value of 0. If a patient survives to the first of discharge or Study Day 29 and is never admitted to an ICU, the number of ICU-free days is 28. If a patient is admitted to an ICU and survives to the first of discharge or Study Day 28, the number of ICU-free days is calculated as 28 minus the number of calendar days from the first ICU admission to final ICU discharge. Days on which the patient was not admitted to an ICU that occurred between days on which the patient was admitted to an ICU do not count towards the number of ICU-free days. Data on ICU use is censored at hospital discharge and the last observed status is carried forward ("in-hospital outcome"). That is, the analysis assumes that a patient who was discharge from the index hospitalization was not readmitted to an ICU between hospital discharge and Study Day 29.

10.9.5 Hospital-free days to Day 28

Hospital-free days to Day 28 is defined as the number of whole calendar days on which the patient was alive and not in the hospital from 00:00 on the day of randomization through Study Day 29. Patients who die before Study Day 29 receive a value of 0. If a patient remains in the hospital during the index hospitalization through Study Day 29, the number of hospital-free days is 0. For patients discharged from the index hospitalization prior to Study Day 28, the number of hospital-free days is calculated as 28 minus the duration of the index hospitalization in calendar days. Readmissions occurring after discharge from the index hospitalization do not contribute to the calculation of the hospital-free days outcome.

11 Definition of Safety Outcomes

11.1 Acute kidney injury

Acute kidney injury is defined according to the creatinine criteria for stage 2 or greater acute kidney injury defined in the 2012 Kidney Disease: Improving Global Outcomes (KDIGO) Clinical Practice Guidelines.[8] Patients who are on renal replacement therapy at the time of randomization will not be eligible for the acute kidney injury outcome. Patients who historically had received renal replacement therapy but were not on renal replacement therapy at the time of randomization are eligible for the acute kidney injury outcome. For determining acute kidney injury, the serum creatinine value closest to randomization will be used as the "baseline" creatinine and creatinine values collected clinically during the patient's index hospitalization will be compared to this baseline. In this trial, urine output will not be used in the definition of acute kidney injury. A patient will be classified as meeting the stage 2 or greater acute kidney injury definition if he/she meets any of the following: (i) creatine value during the index hospitalization at least 2-times the baseline value; (ii) increase in creatinine by an absolute 0.3 mg/dl over baseline and to at least 4.0 mg/dl; initiation of renal replacement therapy.

11.2 Receipt of renal replacement therapy

Patients who are already on renal replacement therapy at the time of randomization will not be eligible for the renal replacement therapy outcome in this trial. Patients who historically had received renal replacement therapy but were not on renal replacement therapy at the time of randomization are eligible for the renal replacement therapy outcome. Patients who have initiation of renal replacement therapy between randomization and hospital discharge will be classified as meeting the renal replacement therapy outcome. Renal replacement therapy includes hemodialysis, peritoneal dialysis and hemofiltration techniques, including continuous veno-venous hemofiltration. Patients who historically had received renal replacement therapy but were not on renal replacement therapy at the time of randomization are eligible for the renal replacement therapy outcome.

11.3 Documented venous thromboembolic disease (DVT or PE)

Patients meet the venous thromboembolism disease outcome if they have a clinically diagnosed deep vein thrombosis (DVT) or pulmonary embolism (PE) between randomization and hospital discharge. The clinical diagnosis must be accompanied by imaging confirmation.

11.4 Documented cardiovascular event (myocardial infarction or ischemic stroke)

Patients meet the cardiovascular event outcome if they have a clinically diagnosed acute myocardial infarction or ischemic stroke between randomization and hospital discharge. A clinical diagnosis of acute myocardial infarction must be accompanied by elevation of the local cardiac troponin assay to greater than 3-times the upper limit of normal. A clinical diagnosis of acute ischemic stroke must be accompanied by imaging confirmation.

11.5 Transfusion reaction

Patients meeting any of the following within 6 hours of initiation of the study product infusion will be classified as experiencing symptoms consistent with a transfusion reaction:

- New increase in body temperature, defined as temperature >38.0 C (100.4 F) or increase in temperature by >1 C (1.8 F) compared to temperature at the beginning of the infusion

- Decline in oxygen saturation, defined as an absolute drop in SpO2 by more than 4% or increase in supplemental oxygen to prevent an absolute drop in SpO2 by more than 4%
- New hives
- New rash (other than hives)
- New itching
- Chest imaging, such as a chest x-ray, demonstrating new or worsening pulmonary edema, pulmonary infiltrates, or effusion
- Clinically diagnosed anaphylaxis

11.6 Transfusion related acute lung injury (TRALI)

Patients meet the TRALI outcome if they have a clinically diagnosed TRALI between initiation of the study product and hospital discharge.

11.7 Transfusion associated circulatory overload (TACO)

Patients meet the TACO outcome if they have a clinically diagnosed TACO between initiation of the study product and hospital discharge.

11.8 Transfusion related infection

Patients meet the transfusion-related infection outcome if they have a clinically diagnosed transfusion-related infection initiation of the study product and hospital discharge.

12 Literature Cited:

1. Royall R. Statistical Evidence: A Likelihood Paradigm. London: Chapman and Hall; 1997.

2. Blume JD. Likelihood methods for measuring statistical evidence. Stat Med 2002;21(17):2563–99.

3. Wald A. Sequential Tests of Statistical Hypotheses. Ann Math Stat 1945;16(2):117–86.

4. Wang S-J, Blume JD. An evidential approach to non-inferiority clinical trials: S.-J. Wang and J. D. Blume. Pharm Stat 2011;10(5):440–7.

5. Polack FP, Thomas SJ, Kitchin N, et al. Safety and Efficacy of the BNT162b2 mRNA Covid-19 Vaccine. N Engl J Med 2020;383(27):2603–15.

6. Cao B, Wang Y, Wen D, et al. A Trial of Lopinavir–Ritonavir in Adults Hospitalized with Severe Covid-19. N Engl J Med 2020;382(19):1787–99.

7. Self WH, Semler MW, Leither LM, et al. Effect of Hydroxychloroquine on Clinical Status at 14 Days in Hospitalized Patients With COVID-19: A Randomized Clinical Trial. JAMA 2020;324(21):2165.

8. Kidney Disease: Improving Global Outcomes (KDIGO), Acute Kidney Injury Work Group. KDIGO clinical practice guideline for acute kidney injury. Kidney Int Suppl 2012;2(1):1.
