# Supplementary material for: Passive Immunity Trial for Our Nation (PassITON): study protocol for a randomized placebo-control clinical trial evaluating COVID-19 convalescent plasma in hospitalized adults
Source: Res Sq. 2021 Mar 2:rs.3.rs-227796. Preprint. [Version 1] doi: 10.21203/rs.3.rs-227796/v1 (PMC7941637; doi:10.21203/rs.3.rs-227796/v1)
Supplement: Supplement [file b95dc5a21ff8e100858dbf45.pptx]

## Slide 1
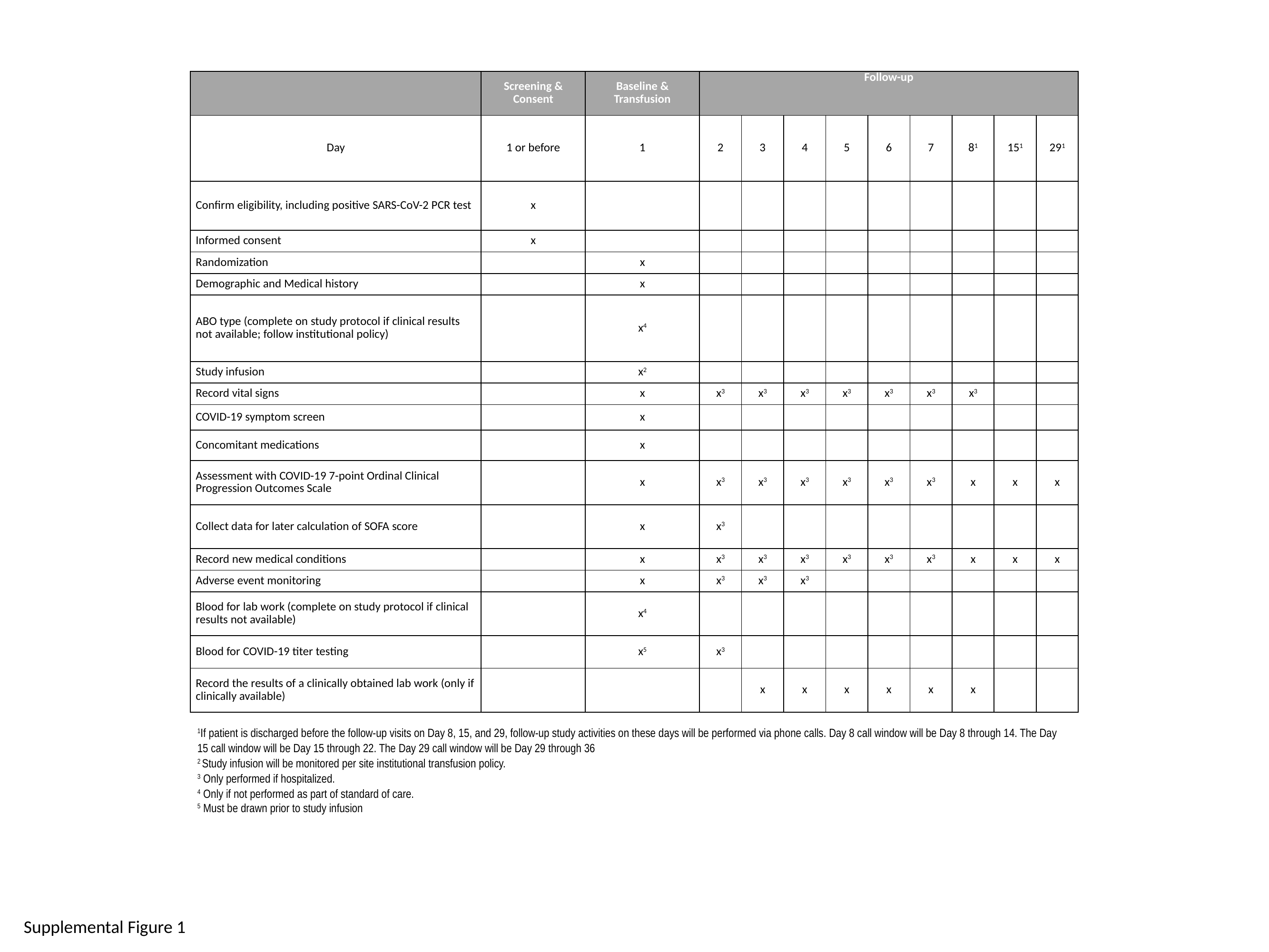

| | Screening & Consent | Baseline & Transfusion | Follow-up | | | | | | | | |
| --- | --- | --- | --- | --- | --- | --- | --- | --- | --- | --- | --- |
| Day | 1 or before | 1 | 2 | 3 | 4 | 5 | 6 | 7 | 81 | 151 | 291 |
| Confirm eligibility, including positive SARS-CoV-2 PCR test | x | | | | | | | | | | |
| Informed consent | x | | | | | | | | | | |
| Randomization | | x | | | | | | | | | |
| Demographic and Medical history | | x | | | | | | | | | |
| ABO type (complete on study protocol if clinical results not available; follow institutional policy) | | x4 | | | | | | | | | |
| Study infusion | | x2 | | | | | | | | | |
| Record vital signs | | x | x3 | x3 | x3 | x3 | x3 | x3 | x3 | | |
| COVID-19 symptom screen | | x | | | | | | | | | |
| Concomitant medications | | x | | | | | | | | | |
| Assessment with COVID-19 7-point Ordinal Clinical Progression Outcomes Scale | | x | x3 | x3 | x3 | x3 | x3 | x3 | x | x | x |
| Collect data for later calculation of SOFA score | | x | x3 | | | | | | | | |
| Record new medical conditions | | x | x3 | x3 | x3 | x3 | x3 | x3 | x | x | x |
| Adverse event monitoring | | x | x3 | x3 | x3 | | | | | | |
| Blood for lab work (complete on study protocol if clinical results not available) | | x4 | | | | | | | | | |
| Blood for COVID-19 titer testing | | x5 | x3 | | | | | | | | |
| Record the results of a clinically obtained lab work (only if clinically available) | | | | x | x | x | x | x | x | | |
1If patient is discharged before the follow-up visits on Day 8, 15, and 29, follow-up study activities on these days will be performed via phone calls. Day 8 call window will be Day 8 through 14. The Day 15 call window will be Day 15 through 22. The Day 29 call window will be Day 29 through 36
2 Study infusion will be monitored per site institutional transfusion policy.
3 Only performed if hospitalized.
4 Only if not performed as part of standard of care.
5 Must be drawn prior to study infusion
Supplemental Figure 1
